# Inter-Patient ECG Heartbeat Classification with Temporal VCG Optimized by PSO

**DOI:** 10.1038/s41598-017-09837-3

**Published:** 2017-09-05

**Authors:** Gabriel Garcia, Gladston Moreira, David Menotti, Eduardo Luz

**Affiliations:** 1 Vale Institute Of Technology, Ouro Preto, MG 35400-000 Brazil; 2 Universidade Federal de Ouro Preto, Computing Department, Ouro Preto, MG 35400-000 Brazil; 3 Universidade Federal do Paraná, Department of Informatics, Curitiba, PR 81531-990 Brazil

## Abstract

Classifying arrhythmias can be a tough task for a human being and automating this task is highly desirable. Nevertheless fully automatic arrhythmia classification through Electrocardiogram (ECG) signals is a challenging task when the inter-patient paradigm is considered. For the inter-patient paradigm, classifiers are evaluated on signals of unknown subjects, resembling the real world scenario. In this work, we explore a novel ECG representation based on vectorcardiogram (VCG), called temporal vectorcardiogram (TVCG), along with a complex network for feature extraction. We also fine-tune the SVM classifier and perform feature selection with a particle swarm optimization (PSO) algorithm. Results for the inter-patient paradigm show that the proposed method achieves the results comparable to state-of-the-art in MIT-BIH database (53% of Positive predictive (+P) for the Supraventricular ectopic beat (S) class and 87.3% of Sensitivity (Se) for the Ventricular ectopic beat (V) class) that TVCG is a richer representation of the heartbeat and that it could be useful for problems involving the cardiac signal and pattern recognition.

## Introduction

Problems related to the rhythm of the heart, called arrhythmias, can be studied and analyzed by inspecting an Electrocardiogram (ECG). To provide an effective treatment for arrhythmias, an early diagnosis is important. Although, the process of identifying and classifying arrhythmias can be tough for a human being because it often demands a thorough analysis for each heartbeat of the ECG signal acquired during hours or even days. Therefore, it is highly desirable to automate the task.

A completely automatic system for arrhythmia classification from ECG signals can be divided into four steps: (1) ECG signal preprocessing; (2) heartbeat segmentation; (3) feature extraction; and (4) learning/classification. The first two steps of such classification system (ECG signal preprocessing and heartbeat segmentation) have been widely explored in the literature^1^. However, there is room for improvements in the steps related to classification (feature extraction and selection).

**Figure 1:**
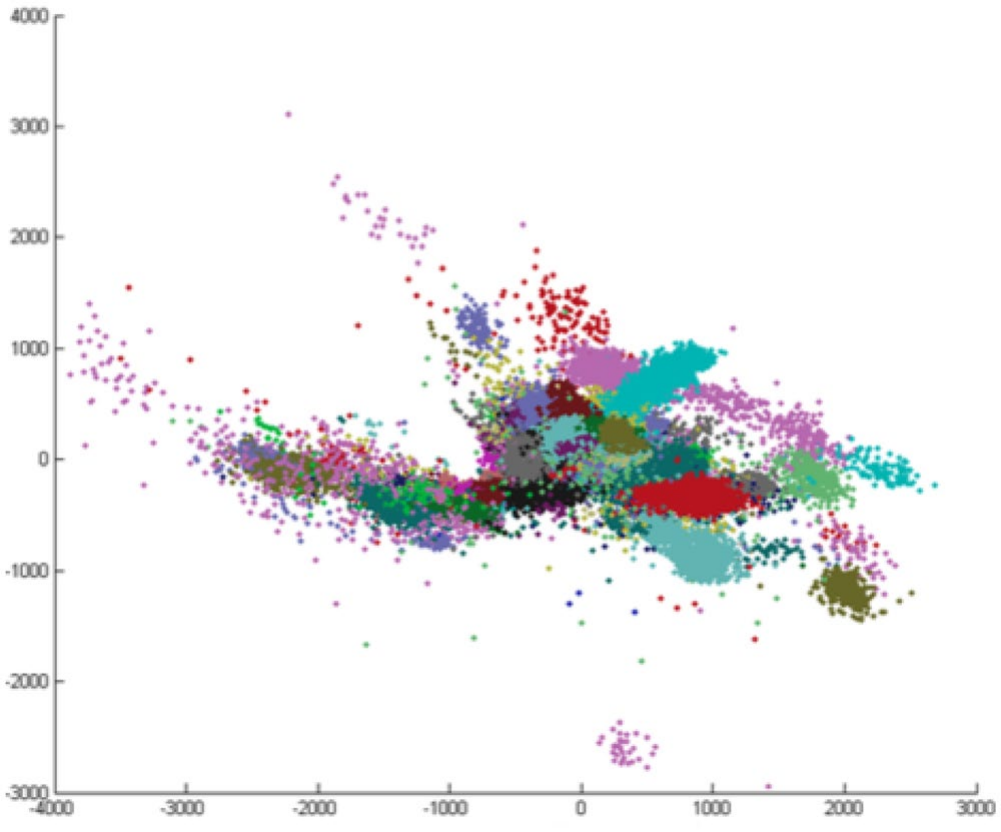
Two principal components of raw ECG heartbeats from patients of MIT-BIH. Each individual is plotted in one color. Adapted^4^.

There are two paradigms for evaluating arrhythmia classification systems^1^: the intra-patient and inter-patient paradigm. In the inter-patient paradigm, heartbeats from a set of individuals are reserved exclusively for method evaluation and heartbeats from different individuals are employed during training of the classification models. In the inter-patient paradigm, classification models do not have contact with heartbeats of individuals from evaluation set. Contrasting to that, for the intra-patient paradigm, both evaluation and train sets have heartbeats from the same individuals. Report results only on the intra-patient paradigm is a serious problem found in the literature since the usage of heartbeats from the same patient for both the training and the testing makes the evaluation process biased^2^. This bias happens because models tend to learn the particularities of the individual’s heartbeats during the training, obtaining expressive numbers during evaluation (very close to 100%) as previously discussed^1–4^. As can be seen in Fig. 1, heartbeats from the same individual tend to be grouped in clusters that, clearly show the individual dependence^4^.

To report results aligned with real world scenarios, it is recommended to follow the Association for the Advancement of Medical Instrumentation (AAMI) standard^5^ and an inter-patient paradigm evaluation protocol such as proposed in ref. 2.

According to the AAMI standard^5^, the Massachusetts Institute of Technology - Beth Israel Hospital (MIT-BIH) Arrhythmia Database is the one recommended for evaluating automatic arrhythmia classification systems. According to AAMI, the heartbeats of this database could be separated into 5 groups or superclasses, and they are: Normal (N), Supraventricular ectopic beat (S), Ventricular ectopic beat (V), Fusion beat (F) and Unknown beat (Q). Many works make use of MIT-BIH Arrhythmia Database, however, few of them follow the AAMI class division scheme and a more realistic evaluation protocol (inter-patient paradigm). Table 1 summarizes recent works on MIT-BIH that evaluate their methods only for the intra-patient paradigm. As one can see in Table 1, it is difficult to assess which technique contribute to arrhythmia classification, since methods with different approaches achieve very high (>98%) accuracies, such as in Chen *et al*.^6^ where 100% accuracy is reported by using only cardiac frequency (RR-interval) information. Such results cannot be taken into consideration from a clinical point of view since the reported values are probably different in a real life scenario concerning accuracy^7^.

**Table 1:** Methods which used the Intra-patient paradigm.

Work	# Classes	Feature set	Classifier	Effectiveness
Cristov & Bortonal, 2004^33^	2	Heartbeat-Intervals, VCG	NN	*Acc* = 99%
Özbay *et al*., 2006^34^	10	Raw-wave	MLP, Fuzzy Cluster, FCNN	*Acc* = 99%
Bortolan *et al*., 2007^35^	2	VCG and Morphological hyperbox + GA	Fuzzy Clustering	*Acc* = 99%
Ubeyli, 2007^36^	4	DWT	SVM, ECOC	*Acc* = 99%
Yu & Chen, 2007^12^	5	ICA, RR-interval	PNN	*Acc* = 99%
Yu & Chen, 2007^12^	6	Wavelet (statistics), RR-interval	PNN	*Acc* = 99%
Minhas & Arif, 2008^37^	6	Wavelet, RR-interval, PCA	kNN	*Acc* = 99%
Asl *et al*., 2008^38^	6	HVR, GDA	SVM	*Acc* = 100%
Chen *et al*., 2014^6^	6	RR-intervals	SVN, NN	*Acc* = 100%
Mert *et al*., 2014^39^	6	RR-intervals, HOS,	Bagged Decision Tree	*Acc* = 99%
			2nd order LPC coeff.	
Alickovic & Subasi, 2015^40^	5	autoregressive (AR) modeling	SVM, MLP, RBF, kNN	*Acc* = 99%
Li *et al*., 2017^41^	6	WPD	GA-BPNN	*Acc* = 99%

Neural Network (NN); Principal Component Analysis (PCA); Generalized Discriminant Analyses(GDA); Error correcting output codes (ECOC); Independent Component Analysis (ICA); Probabilistic neural Network (PNN); Continues Wavelet Transform (CWT); Discrete Wavelet Transform (DWT); Discrete Cosine Transform (DCT); Higher order statistics (HOS); Linear Discriminants (LD); wavelet packet decomposition (WPD). Abbreviations: Acc: Accuracy.

In this work, we present a new heartbeat representation, called the temporal vectorcardiogram (TVCG) and an optimized feature extraction process with complex networks^8, 9^ and particle swarm optimization (PSO). Preliminary results^10, 11^ showed the feasibility of TVCG and complex networks. This work consolidates and extends the approach by introducing the state-of-the-art evolutionary algorithm PSO for feature selection and classifier tuning aiming to improve fully automatic arrhythmia classification. In our view, state-of-the-art methods are presented in Table 2, where all authors follow the same inter-patient paradigm for evaluation as well as AAMI recommendations. For a fair comparison with literature, in this work, the same evaluation approach is followed and our method overcomes state-of-the-art methods, with respect to the Positive predictive (+P) for the Supraventricular ectopic beat (S) class and the Sensitivity (Se) for the Ventricular ectopic beat (V) class.

**Table 2:** Methods which used the Inter-patient paradigm.

Work	Feature set	Classifier	Effectiveness
de Chazal *et al*., 2004^2^	ECG-Intervals, Morphological	Weighted LD	*Acc* = 83%; *Se*_*N*_ = 87%; *Se*_*S*_ = 76%; *Se*_*V*_ = 77% + *P*_*N*_ = 99%; + *P*_*S*_ = 38%; + *P*_*V*_ = 82%
Soria & Martinez, 2009^42^	RR-Intervals, VCG, morphological + FFS	Weighted LD	*Acc* = 90%; *Se*_*N*_ = 92%; *Se*_*S*_ = 88%; *Se*_*V*_ = 90% + *P*_*N*_ = 85%; + *P*_*S*_ = 93%; + *P*_*V*_ = 92%
Llamedo & Martinez, 2011^3^*	Wavelet, VCG + SFFS	Weighted LD	*Acc* = 93%; *Se*_*N*_ = 95%; *Se*_*S*_ = 77%; *Se*_*V*_ = 81% + *P*_*N*_ = 98%; + *P*_*S*_ = 39%; + *P*_*V*_ = 87%
Mar *et al*., 2011^43^	Temporal Features, Morphological, statistical features + SFFS	Weighted LD MLP	*Acc* = 89%; *Se*_*N*_ = 89%; *Se*_*S*_ = 83%; *Se*_*V*_ = 86% + *P*_*N*_ = 99%; + *P*_*S*_ = 33%; + *P*_*V*_ = 75%
Ye *et al*., 2012^44^	Morphological, Wavelet, RR interval, ICA, PCA	SVM	*Acc* = 86.4% *Se*_*N*_ = 88%; *Se*_*S*_ = 60%; *Se*_*V*_ = 81% + *P*_*N*_ = 97%; + *P*_*S*_ = 53%; + *P*_*V*_ = 63%
Lin & Yang, 2014^45^*	normalized RR-interval morphological features	weighted LD	*Acc* = 93%; *Se*_*N*_ = 91%; *Se*_*S*_ = 81%; *Se*_*V*_ = 86% + *P*_*N*_ = 99%; + *P*_*S*_ = 31%; + *P*_*V*_ = 73%
Huang *et al*., 2014^46^**	Random projection RR-intervals	Ensemble of SVM	*Se*_*N*_ = 99%; *Se*_*S*_ = 91%; *Se*_*V*_ = 94% + *P*_*N*_ = 95%; + *P*_*S*_ = 42%; + *PV* = 91%

Artificial Neural Network (ANN); Principal Component Analysis (PCA); Floating Feature Selection (FFS); Independent Component Analysis (ICA); Back Propagation Neural Network (BPNN); Linear Discriminants (LD); Sequential forward floating search (SFFS); *Authors optimize their result for 3 classes (N), (S), (V); **Where confusion matrix was not given, some values could not be computed. Abbreviations: (N): Normal heartbeat; (S): Supraventricular ectopic heartbeat; (V): Ventricular ectopic heartbeat; Acc: Accuracy; +P: Positive predictive; Se: Sensitivity.

## Results

### Database setup

Experimental analysis is conducted with the MIT-BIH database, following the inter-paradigm protocol proposed by de Chazal^2^: the database is split into two groups: DS1 and DS2. DS1 is used for training and DS2 for evaluation.

In order to perform the evolution of the system in an unbiased way, it is also important to use different data for the evaluation in the optimization stages. For this, the DS1 group was divided into two new subgroups, DS11 and DS12, for training and evaluation, respectively. In agreement with the suggestions of Luz *et al*.^1^, in both separations, the heartbeats of each patient remained in only one group. The groups are divided seeking a balance in the representation of classes. In Table 3 records partitions are shown, as well as the number of heartbeats for each class. Also, to comply with the AAMI standard^5^, four records related to patients wearing an electronic pacemaker are disregarded.

**Table 3:** Records used and number of representatives of each class for each of the partitions.

Partition	Registers	Class (N)	Class (S)	Class (V)
DS1	101, 106, 108, 109, 112, 114, 115, 116, 118, 119, 122, 124, 201, 203, 205, 207, 208, 209, 215, 220, 223, 230	45543	782	3469
DS11	101, 106, 108, 109, 114, 115, 116, 119, 122, 209, 223	22249	474	1615
DS12	112, 118, 124, 201, 203, 205, 207, 208, 215, 220, 230	23294	308	1854
DS2	100, 103, 105, 11, 113, 117, 121, 123, 200, 202, 210, 212, 213, 214, 219, 221, 222, 228, 231, 232, 233, 234	44049	1808	3143
Total		89592	2590	6612

### Metric evaluation

The metrics recommended by AAMI for arrhythmia classification methods are: Sensitivity (Se), Positive predictive (+P), False positive rate (FPR) and Overall accuracy (Acc). Overall accuracy can be strongly distorted by majority class figures. Therefore, the first three metrics are the most relevant for comparing the methods, since the classes for heartbeat types are extremely imbalanced in MIT-BIH database.

Fig. 4 illustrates metric calculation definitions. Note that in sections (a), (b), and (c) of Fig. 4, formulas and schemes to compute Se, +P, FPR and Acc are given for the V, S and N classes, respectively.

### Proposed Method Flow

The proposed method flow is depicted in Fig. 2.

1. **Complex network optimization** - TVCG is an electrical activity in a 3D-plot, wherein two leads are considered, MLII and V1, along with time as a new axis. From this 3D-plot (See Fig. 3) features for the classification step are extracted using complex networks^8, 9^. The complex networks parameters are estimated by the state-of-the-art evolutionary algorithm called Particle Swarm Optimization (PSO). The PSO’s task is to find best complex network parameter combination (T_0_, T_Q_ and *m*), in the range from 1 to 10; 0.001 to 0.2; 0.1 to 1, respectively. To accomplish this, PSO parameters are defined as: 100 particles and 50 generations. At first stage, TVCG features from the complex network are combined with the RR interval and fed to the SVM classifier, with fixed weights (*w*1 = 6, *w*2 = 100 and *w*3 = 15) to compensate database imbalance^10^. For this and the two next optimization processes, the training was carried out in DS11 and the tests in DS12, and all data was used without any preprocessing filter. The best complex network parameters are used in the following step.

**Figure 2:**
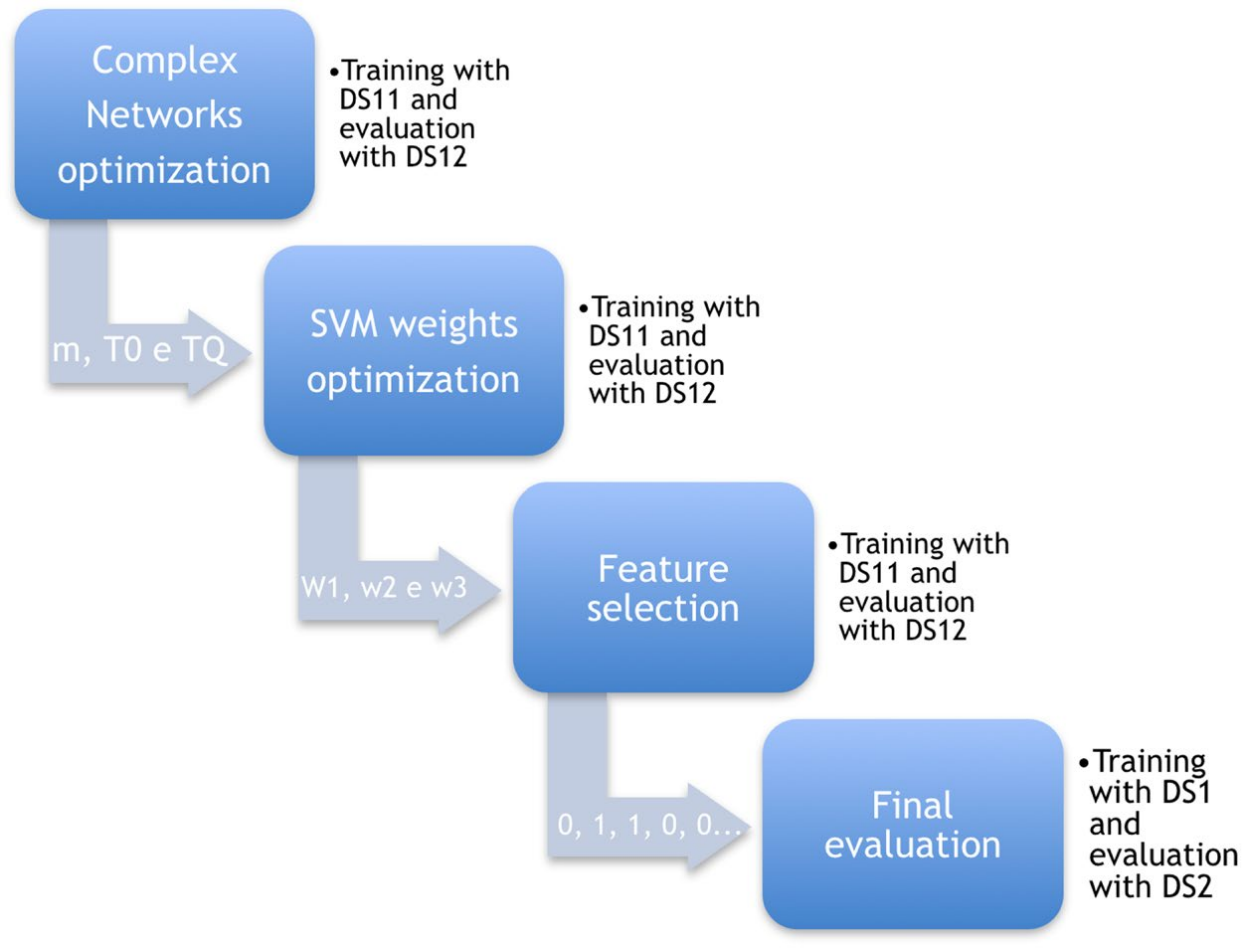
Proposed method flow.
2. **SVM weights optimization** - Once TVCG features are extracted, two other sets of features are included in the feature vector, morphological features^2^, and wavelet based features^12^, resulting in a vector of size 178. Those features are known to be efficient for arrhythmia classification problem and they are considered here to promote competition in the feature selection stage. The 178 dimension feature vector is used to fine-tune SVM weights and for this task, the PSO is executed with 100 individuals and 100 generations, and the objective is to search for the best configuration of *w*1, *w*2 and *w*3 in the range of 1 to 5; 10 to 200; 5 to 100, respectively. The SVM is configured with RBF kernel, and hard margin (*C* = 1 and $\gamma =\frac{1}{{\rm{number}}\,{\rm{of}}\,{\rm{features}}}$).
3. **Feature selection** - With the tuned SVM, a wrapper feature selection is performed. Evaluating all possible feature vector combinations would be unfeasible. Thus, a PSO is used to reduce computational cost. In this case, binary PSO is employed (BPSO). The BPSO initial population is setup to include all features of the vector and then gradually reduces the vector size. The BPSO is executed during 100 iterations with 300 particles.
  The best feature vector combination, according to feature selection algorithm proposed here, includes 64 features from a list of 178. Among them, 50 morphological^2^, 5 from ECG intervals^2^, 4 statistical features calculated on wavelet coefficients^12^ and all 5 features from TVCG.
4. **Final evaluation** - The best SVM model selected in the last step is considered for final evaluation. The final evaluation is performed with training (DS1) and test (DS2) datasets on three different preprocessings: No Filter, de Chazal Filter and Common Filter.

**Figure 3:**
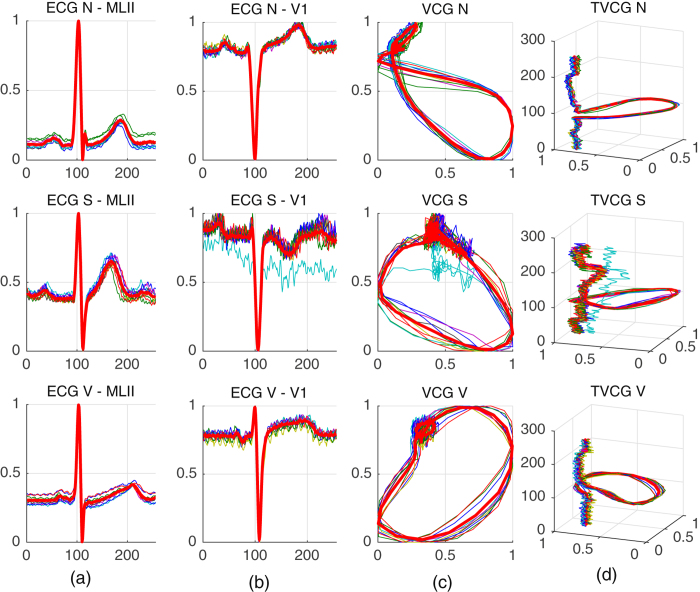
(**a**) Lead A (MLII); (**b**) Lead B (V1); (**c**) VCG; (**d**) TVCG. Ten heartbeats and mean heartbeat of 3 classes (N, S and V) from records 116, 215 and 220 of the MIT-BIH.

**Figure 4:**
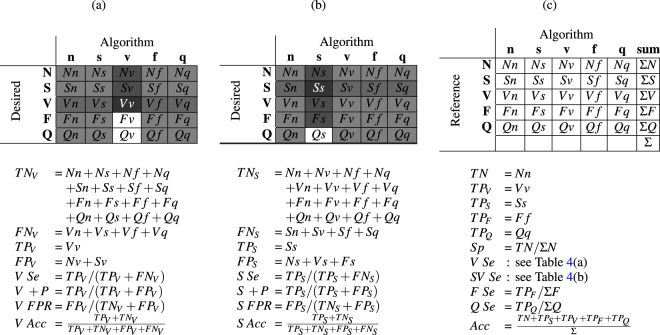
Calculations for metric evaluations. (a), (b), and (c) highlight the calculation of metrics for V, S, and N, respectively. Source^2^. Abbreviations: Acc: Accuracy; F: Fusion heartbeat group (superclass); FPR: False positive rate; *N*: Normal heartbeat group (superclass); +P: Positive predictivity; Q: Unknown heartbeat group (superclass); Se: Sensitivity; Sp: Specificity; S: Supraventricular ectopic heartbeat group (superclass); V: Ventricular ectopic heartbeat group (superclass); TN: True negative; and TP: True positive.

## Discussion

As can be seen in Table 4, preprocessing has a great impact on final classification and more attention should be given in the literature to the preprocessing stage. To the best of our knowledge, there is no systematic study of different filters/pre-processing techniques, following an inter-patient scheme and the AAMI recommendations. Such a study could shed light on how the filtering process impact the classification of arrhythmic classes.

**Table 4:** Test results (DS2) of the best parameters configuration.

Filters	Acc	Class (N)			Class (S)			Class (V)		
		Se	+P	FPR	Se	+P	FPR	Se	+ P	FPR
de Chazal	85,8	87,6	96,6	27,6	28,7	24,4	3,4	92,6	42,2	8,7
Common	92,4	94,0	98,0	17,4	62,0	53,0	2,1	87,3	59,4	4,1
Without filter	83,1	84,1	97,1	22,6	50,8	13,0	13,1	88,6	74,0	2,1

Se, +P and FPR stands for Sensitivity, Positive Predictive Value and False Positive Rate, respectively.

As shown in Table 5, the proposed method achieves results comparable to state-of-the-art methods, with a global accuracy of 92.4% when Common filters are applied. In Table 5 we also show the result of the proposed methodology with conventional VCG. As can be seen in Fig. 3(c) and (d), the time axis allows the signal to be disentangled and therefore offers more information. With the TVCG, the differentiation of the heartbeats type is more apparent. The heartbeats of class N are less confused to other classes and thus, the positive predictive is higher with TVCG as well as the false positive rate (See Table 6). The excessive false alarm is a major problem for clinical use since it diminishes operator confidence in the algorithm/equipment. As one can see in Table 5, our method with TVCG has a more balanced positive predictive rate, compared to the literature.

**Table 5:** Compared methods which used inter-patient paradigm and three classes (N), (S) and (V) for classification.

Work	Acc	Class (N)			Class (S)			Class (V)		
		Se	+P	FPR	Se	+P	FPR	Se	+P	FPR
Proposed method	92.4	94.0	98.0	17.4	62.0	53.0	2.1	87.3	59.4	4.1
Proposed method on VCG	78.0	79.1	96.3	27.0	31.2	8.4	13.0	89.5	46.1	7.2
Lin & Yang, 2014^45^	93.0	91.0	99.0	—	81.0	31.0	—	86.0	73.0	—
Llamedo & Martínez, 2011^3^	93.0	95.0	98.0	—	77.0	39.0	—	81.0	87.0	—
Garcia et. al, 2016^10^	91	95	96	28	30	26	3	85	66	3

**Table 6:** Confusion matrix for best parameters configurations, Common Filter in Table 4.

	Predicted Classes		
	(n)	(s)	(v)
**True Classes**			
(N)	41043	792	1834
(S)	657	1111	25
(V)	200	195	2724

The proposed feature selection aims at reducing the size of the feature vector in order to reduce the computational cost and increase the generalization power of the method. Also, the BPSO objective function maximizes the F-score metric, tending to promote a balance between Se and +P of the classes. Results showed that PSO is an outstanding tool for feature selection in the arrhythmia classification problem. In this context, all five features extracted with complex networks from TVCG have been chosen.

Results presented herein corroborate the efficiency of the new ECG representation proposed in this work, TVCG, along with the complex network’s method for feature extraction. However, the analysis has two limitations due to the MIT-BIH database. According to Schulte-Frohlinde *et al*.^13^, some types of arrhythmias may have an intrinsic periodicity and long-term analysis (12 to 24 hours) is desirable to investigate these patterns^14^. Though MIT-BIH database has records of only a 30 minutes duration which hinders the investigation of patterns associated to long term periodicity. Only a few studies have considered the dynamical properties of the ECG in detail for the long term, and the lack of a benchmark database could be the problem. The MIT-BIH database is also limited regarding the number of leads. Currently, medical diagnostic systems make use of 12 leads and new databases, that cover these issues, should be proposed in the literature and recommended by AAMI. Time series forecasting models^15^ could provide interesting insight on modeling the dynamical arrhythmias features of the ECG signal and could be an promising research path.

The new heartbeat representation could be further explored in the literature and a promising direction would be to explore the TVCG for QRS detection and detection of other ECG fiducial points since it is a richer view of the heartbeat signal. TVCG would also be suited to other data representation methods such as based on Deep Learning, that today represents the state-of-the-art data representation methods on several machine learning and computer vision tasks^16^.

## Methods

In this work a novel heartbeat representation is explored, the temporal vectorcardiogram (TVCG), along with an efficient feature extraction technique for TVCG called Complex Networks^10^. In addition to that, an optimization stage based on particle swarm optimization is employed to select best features and fine-tune the classifier. Also, two popular preprocessing techniques are evaluated on a popular benchmark database.

### MIT-BIH Database

The MIT-BIH database was created to be representative and provide real clinical situations/scenarios. This database is presented in majority of the publications found in the literature. The MIT-BIH database consists of 48 annotated records obtained from 47 patients studied by the Beth Israel Hospital Arrhythmia Laboratory in Boston, USA, between 1975 and 1979. Each record has 30 minutes ECG acquisition of two leads sampled at 360 Hz. In total, the database has more than 109.000 heartbeats and each one is labeled as a heartbeat type. In the majority of the records the principal lead (lead A) is a modification of lead II (electrodes on the chest). The other lead (lead B) is usually lead V1, modified, but in some records, this lead is known to be V2, V5 or V4. Generally, lead A is used to detect heartbeats, since the QRS complex is more prominent in this lead. Lead B favors the arrhythmic classification of the types SVEB and VEB^17^. More information regarding this database can be found in ref. 18.

### Preprocessing

In order to improve the quality of signals from the database and to remove excessive noise, preprocessing techniques could be used. Two preprocessing methods are applied and evaluated.

The first preprocessing method is proposed by de Chazal^2^ and contemplates two median filters (200-ms, 600-ms), baseline removal and a 12 taps low-pass filter with 3 dB at 35 Hz^2^.

The second preprocessing is proposed by Queiroz *et al*.^11^, called Common Filter, and it is composed of two finite impulse response (FIR) filters. The first one is a 12 tap low pass filter with −3 dB at 35 Hz, while the second one is a high-pass filter with −3 dB at 1 Hz.

There is substantial evidence in the literature supporting the idea that the ECG signal has a fractal temporal structure^19–21^. Therefore the signal dynamics could be related to intrinsic properties of heart control mechanisms. Although, it could be difficult to differentiate, by looking at the ECG signal, what would be generated by external interference from components generated by the intrinsic heart control mechanism. To avoid accidentally filtering an important signal component, experiments are also performed with raw data (without the filtering process).

### Feature Extraction

Some major contribution of this work are the novel heartbeat representation, the TCVG, and an efficient feature extraction for the TVCG, based on complex networks. To promote competition during the feature selection, state-of-the-art features are also considered and detailed here: morphological features^2^ and first order statistics along with wavelet coefficients^12^.

#### Temporal Vectorcardiogram and complex networks

The Vectorcardiogram (VCG) is a two-dimensional representation of the ECG that uses the signal of two distinct leads. Each lead is used as an axis of a 2D plot. The VCG representation was used for feature extraction in previous works (Llamedo and Martínez^3^ and Queiroz *et al*.^11^), presenting promising results. Nonetheless, the VCG representation discards time information, i.e, it loses the time correlation between samples. This work proposes the use of a new ECG representation method, which is based on VCG but also considers time as a third dimension, enriching conventional VCG and allowing the extraction of more relevant features for the arrhythmia classification task. Using the two ECG leads plus the time, a three-dimensional ECG representation is built, called Temporal Vectorcardiogram (TVCG) by the authors. In Fig. 3, there can be seen a heartbeat from three different representations, ECG, VCG and TVCG.

The complex networks theory is an intersection of two main areas, the graph theory and statistical analysis. In this work, the using of complex networks to extract features from the TVCG is proposed. To accomplish this the TVCG is considered as a set of points, $V=[{p}_{1},{p}_{2},\ldots ,{p}_{{n}_{v}}]$, where each point *p*_*i*_ = [*x*_*i*_, *y*_*i*_, *z*_*i*_] is a vector with components *x*_*i*_, *y*_*i*_, *z*_*i*_ representing the two leads and the time, respectively. The network is constructed by considering each point *p*_*i*_ a vertex and the euclidean distance between each pair of points *d*(*p*_*i*_, *p*_*j*_) defined as the weight of each edge *e*_*i*,*j*_ between these two points. The network corresponds to the graph *G* = (*V*, *E*), wherein *E* = {*e*_*i*,*j*_|*i*, *j*∈{1, …, *nv*}} and *V* are the set of edges and vertices. Finally, a square matrix *W* = (*w*_*ij*_) is obtained, of order *n*_*v*_, i.e.

1 $${w}_{ij}=\sqrt{{({x}_{i}-{x}_{j})}^{2}+{({y}_{i}-{y}_{j})}^{2}+{({z}_{i}-{z}_{j})}^{2}},$$

wherein *i*, *j* ∈ {1, …, *n*_*v*_}. The next step, then, is the normalization of these values for the interval [0,1]. At this point, the graph is just a regular network, whereas all vertices are connected to each other. To make a complex network from our regular network, the dynamic evolution method is applied, where in each iteration, the vertices with weight *w*_*ij*_ greater than a limit *T*_*l*_ are removed from the network. Doing this, we have the complex network. From that complex network, five features are extracted based on their characteristics. To extract these features some variables are needed to be defined: the initial limit *T*_0_, the final limit *T*_*Q*_ and the total number of iterations *m*. The limit *T*_*l*_ of the actual iteration is calculated by:

2 $${T}_{l}={T}_{0}+l\frac{({T}_{Q}-{T}_{0})}{(m-1)},$$

wherein *T*_0_, *T*_*Q*_ and *m* will be defined in the optimization process^9^. Following that which has been previously proposed in the literature^9^, the five features extracted from the complex network are:

1. Mean connectivity degree.
2. Maximum connectivity degree.
3. Joint degree entropy.
4. Joint degree energy.
5. Mean joint degree.

The first two features are related to the number of vertices connected to each other and the other related to probabilities of a vertex being connected to another. Therefore, a vector with *m* × 5 features is used to feed the classifiers.

#### Morphological features

Many features can be extracted with the aid of ECG fiducial points. The following features^2^ are considered state-of-the-art in the literature and also considered here:

- RR-Interval: The R-point is often used to obtain information about the cardiac rhythm. Three features are calculated using the RR interval (the time between two R points), these are: pre-RR, the interval RR between the heartbeat concerned and its previous one; post-RR, between the concerned and its posterior one; and the local average, which is the RR interval average of the intervals surrounding the heartbeat.
- Heartbeat Interval: three features are extracted per each lead, relative to: QRS complex duration, calculated between the beginning and the end of the QRS; T wave duration, calculated between the end of the QRS and the beginning of the T wave; and the presence or absence of the P-wave in the heartbeat. The beginning and end of QRS and T waves was detected by running ecgpuwave software (Source at https://physionet.org/physiotools/ecgpuwave/ **)**.
- ECG morphological: A set of features extracted directly from ECG wave morphology: 10 samples between the QRS complex; 9 samples between the QRS and the T wave start; 10 samples between the FP-50ms and FP + 100ms; 8 samples between the FP + 150ms e FP + 500ms. A more detailed description can be seen in (*de Chazal et al., 2004*, Fig. 3)^2^. Both the normalized and absolute values of these features were used.

#### Wavelet and the autocorrelation function

The discrete wavelet transform has been widely used for signal processing in the last decades. Since the ECG signal is highly irregular and non-stationary, Wavelet transform can filter dominant features related to non-stationaries^22^ and could favors the appearance of hidden but important features for arrhythmia classification. Also, Wavelet transform preserves the Fourier phase information^22^. In this work, following the proposed in ref. 12, we applied the Haar Wavelet twice on lead A, getting the approximation A2 and the details D1 and D2. The autocorrelation was used to extract features from the three sub-bands resultant from the Discrete Wavelet Transform (DWT). A technique used to find repetitive patterns in signals, it can be considered as a measurement of the coherence between the signal *x*(*n*) and its shifted version. If *x*(*n*) has a size *N*, the autocorrelation function is expressed as

$$A{C}_{x}(l)=\sum _{n=i}^{N-|k|-1}x(n)x(n-l)$$

wherein *l* is the time shift index, *i* = *l*, *k* = 0 for *l* ≥ 0 and *i* = 0, *k* = *l* for *l* < 0. As used in ref. 12, for the experiments we used *l* = 1. Other feature groups extracted from the DWT sub-bands are the relative amplitudes, calculated as $\frac{\min (x(n))}{\max (x(n))}$. Despite it being in this section because it came from the wavelet, the relative amplitudes can be considered as a morphological feature. Yu and Chen^12^ proposed using the variance of the QRS samples as another morphological feature.

### Classifier

SVM^23^ is a very popular classifier in the literature for ECG-based arrhythmia classification methods. The SVM is a technique based on the principle of *structural risk minimization* ^24^ aiming at establishing a separating function between two classes depending on input.

3 $$f:{\mathscr{X}}\to \{\pm 1\},$$

Vapnik^24^ proposed hyperplanes in a dot product space ${\mathcal H}$, and also the *Generalized Portrait* learning algorithm for problems that can be separated by hyperplanes.

4 $$\langle {\bf{w}},{\bf{x}}\rangle +b=\mathrm{0,}$$

wherein ${\bf{w}},{\bf{x}}\in  {\mathcal H} ,b\in {\mathbb{R}}$, correspond to the decision function:

5 $$f(x)={\rm{s\; gn}}(\langle {\bf{w}},{\bf{x}}\rangle +b),$$

Aiming at that, Vapnik^24^ first considered that there must exists a unique *optimal hyperplane* distinguished by the maximum margin of separation between any training point and the hyperplane. Second, the over-fitting of the separating hyperplanes decreases with an increasing margin.

Therefore, to achieve the optimal hyperplane, it is necessary to solve:

6 $$\mathop{{minimize}}\limits_{{\bf{w}}\in  {\mathcal H} ,b\in {\mathbb{R}}}\quad \tau ({\bf{w}})=\frac{1}{2}{\Vert {\bf{w}}\Vert }^{2},$$

subject to:

7 $${y}_{i}(\langle {\bf{w}},{{\bf{x}}}_{i}\rangle +b)\ge 1\quad {\rm{for\; all}}\quad i=1,\mathrm{...},m,$$

in which the constraint in eq:constr should ensure that *f*(*x*_*i*_) will be +1 for *y*_*i*_ = +1 and −1 for *y*_*i*_ = −1, and also the scale of ***w*** should be fixed. A more detailed discussion of these parameters is provided by Schölkopf and Smola^25^.

The *τ* function in eq:mini is the *objective function*, and the *inequality constraints* are the ones in eq:constr. Thus, they form a *constrained optimization problem*. The separating function is a weighted combination of elements of the input (training dataset). These elements are named as *Support Vectors* and determine the boundary between classes.

In order to cope with possible nonlinearities on input data, the *kernel trick* could be used to improve performance^25^. Although, some examples could violate eq:constr and to allow flexibility, the slack variables *ξ* ≥ 0 are introduced^25^, which leads to the constraints:

8 $${y}_{i}(\langle {\bf{w}},{{\bf{x}}}_{{\bf{i}}}\rangle +b)\ge 1-{\xi }_{i}\quad {\rm{forall}}\quad i=1,\mathrm{...},m.$$

Controlling both the margin (through ||***w***||) and slack variables ∑_*i*_ *ξ*_*i*_ is then possible to find a classifier that efficiently generalizes by minimizing the objective function:

9 $$\tau ({\bf{w}},\xi )=\frac{1}{2}{\Vert {\bf{w}}\Vert }^{2}+C\sum _{i=1}^{m}{\xi }_{i},$$

subject to the constraint in eq:slconst2, in which the constant *C* > 0 determines a trade off between over-fitting and generalization. SVM approaches that rely on the tuning variable C are called C-Support Vector Classifiers (C-SVC)^23^.

In this work the well known libSVM^26^ is employed. The libSVM implementation provides a multi-class C-SVC by means of an *one-against-one* approach, in which for *k* classes, *k*(*k* − 1)/2 classifiers are trained for a pair of classes. The libSVM also provides a way to compensate the class imbalance by using different C constant values for each class.

### Optimizing the complex networks and SVM

A problem encountered many times when using techniques such as complex networks, which have several parameters and whose efficiency depends on the correct choice of their values, is the difficulty in finding such parameters efficiently. In this proposed method, besides the values of *T*_0_, *T*_*Q*_ and *m* of the complex networks, there are the weights *w*1, *w*2 and *w*3, corresponding to the weights of classes N, SVEB and VEB, respectively, used in the SVM to try to balance these classes. To find the best parameters and use the tools effectively, an optimization technique called particle swarm was applied.

#### Particle swarm optimization

Particle Swarm Optimization (PSO) is an evolutionary algorithm based on group behavior, such as the movement of flocks, or shoals. It was first introduced in 1995 by Kennedy & Eberhart (1995)^27^. The PSO is similar to a genetic algorithm where the system is initialized with an initial population, treated in the PSO as a set of particles, each particle being a possible solution to the problem. From this initial population, a new population is generated at each iteration according to a function, called velocity, which is calculated based on the position of the best generation particle (called gbest) and the best position of the individual in question (called pbest). The idea is that this movement of particles in the space of solutions, in the sense of gbest and pbest, will cause each generation to move towards the optimum solution, simultaneously realizing a global search and a local search. In Settles (2005) there is a summary on the functioning of the PSO and variations of the algorithm are presented. For this work, we used the PSO with an inertia factor, which causes the algorithm to reduce the speed increase with the iterations so that the PSO starts to focus more on a local search at the end of its execution. Algorithm 1 shows the pseudo code of the algorithm used.

The velocity of the particles is calculated by:

10 $${v}_{i}^{k}(t)=w(t)\ast {v}_{i}^{k}(t-1)+{c}_{1}{\gamma }_{1i}({{\rm{p}}{\rm{b}}{\rm{e}}{\rm{s}}{\rm{t}}}_{i}^{k}-{x}_{i}^{k}(t-1))+{c}_{2}{\gamma }_{2i}({{\rm{g}}{\rm{b}}{\rm{e}}{\rm{s}}{\rm{t}}}_{i}^{k}-{x}_{i}^{k}(t-1))$$

wherein ${v}_{i}^{k}(t)$ is the *i* th component of the velocity of the *k* th particle, ${x}_{i}^{k}(t)$ is the *i* th component of the position of the *k* th particle, in the *t* th step of the algorithm. The external parameters defined are: *w*(*t*) is the inertia weight, with decreased linearly from about 0.9 to 0.4 during a run; the acceleration constants *c*_1_ and *c*_2_, are usually set at 2.05^28^; *γ*_1_ and *γ*_2_ represent a positive random number with uniform distribution between 0 and 1.

From the velocity and the previous position of the particle its new position is calculated:

11 $${x}_{i}^{k}(t)={x}_{i}^{k}(t-1)+{v}_{i}^{k}(t)$$

The *fitness* used to evaluate the classification was the F-score^29^, that is a measure of a test’s accuracy based the harmonic mean of the Sensitivity and Positive Predictive, calculated by

12 $$F-score=2\ast \frac{Se\ast (+P)}{Se+(+P)},$$

Since for each class an F-score is calculated, fitness is the arithmetic mean of the F-score of each of the three classes. A weighted mean can be used to prioritize the results of one class or another during evolution.

**Algorithm 1:**
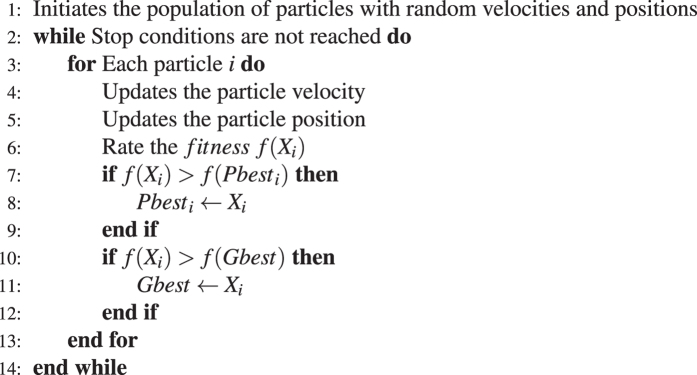
PSO with inertia factor.

### Feature Selection

This work aims to use features proposed in three different works, making the feature vector dimension quite high. Given the behavior of the classifier used, the SVM, characteristics that have little separability relationship between the classes can considerably decrease the performance of the classifier. Therefore, the selection of the most relevant characteristics for classification becomes an important step and may result in a significant increase in overall accuracy.

The groups of characteristics and their respective sizes, separated by the authors that propose them, are:

- Morphological Features^2^, - Results in 161 characteristics, of which 74 are extracted directly from the raw wave of both leads and another 13 from information about the fiducial points.
- Complex networks applied to TVCG^10^, - They can vary their size according to the number of iterations *m*, with each iteration generating 5 characteristics.
- Wavelet and autocorrelation function^12^, - Two values are extracted from each of the 3 bands added to a characteristic of the QRS complex, totaling 7 features.

Considering the use of all characteristics presented, we have a feature vector with a dimension equal to or greater than 173.

#### Binary PSO

The particle swarm optimization algorithm allows applications in binary problems,^30^. The characteristic selection is a binary type problem, where one wants to find a vector of zeros and ones, saying whether or not the characteristic should be used. The binary PSO (BPSO) was proposed with the aim of finding the best characteristics in some works in the literature, as in refs 31, 32, in both cases having presented good results.

The proposed BPSO approach for feature selection problem basically follows the execution of the sequence of steps of the algorithm 1, update the position of the particle according to Equation 13:

13 $${x}_{i}^{k}=\{\begin{array}{cc}1, & {\rm{r}}{\rm{a}}{\rm{n}}{\rm{d}}() < s({v}_{i}^{k})\\ 0 & {\rm{o}}{\rm{t}}{\rm{h}}{\rm{e}}{\rm{r}}{\rm{w}}{\rm{i}}{\rm{s}}{\rm{e}}\end{array}$$

wherein $s({v}_{i}^{k})=\frac{1}{1+{\exp }^{-{v}_{i}^{k}}}$, and rand() positive random number whose uniform distribution is between [0; 1.0]. In feature selection phase, we tested empirically the BPSO with linear decreasing inertia weight and no inertial weight. The BPSO with no inertial weight resulted in a better F-score compared when the inertial weight was used.

## References

[1] Luz EJdS, Schwartz WR, Cámara-Chávez G, Menotti D (2016). Ecg-based heartbeat classification for arrhythmia detection: A survey. Computer methods and programs in biomedicine.

[2] de Chazal P, O’Dwyer M, Reilly RB (2004). Automatic classification of heartbeats using ECG morphology and heartbeat interval features. IEEE Transactions on Biomedical Engineering.

[3] Llamedo M, Martnez JP (2011). Heartbeat classification using feature selection driven by database generalization criteria. IEEE Transactions on Biomedical Engineering.

[4] Nunes, T. M. *Classificação de arritmias cardacas em eletrocardiograma utilizando floresta de caminhos ótimos*. Ph.D. thesis (2014).

[5] ANSI/AAMI. Testing and reporting performance results of cardiac rhythm and ST segment measurement algorithms. American National Standards Institute, Inc. (ANSI), Association for the Advancement of Medical Instrumentation (AAMI), ANSI/AAMI/ISO EC57, 1998-(R)2008 (2008).

[6] Chen H, Cheng B-C, Liao G-T, Kuo T-C (2014). Hybrid classification engine for cardiac arrhythmia cloud service in elderly healthcare management. Journal of Visual Languages & Computing.

[7] Luz, E. & Menotti, D. How the choice of samples for building arrhythmia classifiers impact their performances. In *Engineering in Medicine and Biology Society, EMBC, 2011 Annual International Conference of the IEEE*, 4988–4991 (IEEE, 2011). 10.1109/IEMBS.2011.6091236 22255458

[8] Watts DJ, Strogatz SH (1998). Collective dynamics of ‘small-world’ networks. Nature.

[9] Backes AR, Casanova D, Bruno OM (2009). A complex network-based approach for boundary shape analysis. Pattern Recognition.

[10] Garcia, G., Luz, E., Moreira, G. & Menotti, D. Improving automatic cardiac arrhythmia classification: Joining temporal-vcg, complex networks and svm classifier. In *International Joint Conference on Neural Networks (IJCNN 2016)* (IEEE, 2016).

[11] Queiroz, V., Luz, E., Moreira, G., Guarda, A. & Menotti, D. Automatic cardiac arrhythmia detection and classification using vectorcardiograms and complex networks. In *Engineering in Medicine and Biology Society (EMBC), 2015 37th Annual International Conference of the IEEE*, 5203–5206 (2015). 10.1109/EMBC.2015.7319564 26737464

[12] Yu S-N, Chen Y-H (2007). Electrocardiogram beat classification based on wavelet transformation and probabilistic neural network. Pattern Recognition Letters.

[13] Schulte-Frohlinde V (2001). Noise effects on the complex patterns of abnormal heartbeats. Phys. Rev. Lett..

[14] Schulte-Frohlinde V (2002). Complex patterns of abnormal heartbeats. Physical Review E.

[15] Coelho, I. M. *et al*. A gpu deep learning metaheuristic based model for time series forecasting. *Applied Energy* (2017).

[16] LeCun Y, Bengio Y, Hinton G (2015). Deep learning. Nature.

[17] Goldberger AL (2000). Physiobank, physiotoolkit, and physionet: Components of a new research resource for complex physiologic signals. Circulation.

[18] Moody, G. B. & Mark, R. G. The mit-bih arrhythmia database on cd-rom and software for use with it. In *Computers in Cardiology 1990, Proceedings*., 185–188 (IEEE, 1990).

[19] Ivanov PC (1999). Multifractality in human heartbeat dynamics. Nature.

[20] Amaral LAN (2001). Behavioral-independent features of complex heartbeat dynamics. Physical Review Letters.

[21] Ivanov PC (2001). From 1/f noise to multifractal cascades in heartbeat dynamics. Chaos: An Interdisciplinary Journal of Nonlinear Science.

[22] Ivanov PC, Rosenblum MG, Peng C-K, Mietus J (1996). Scaling behaviour of heartbeat intervals obtained by wavelet-based time-series analysis. Nature.

[23] Cortes C, Vapnik V (1995). Support vector networks. Machine Learning.

[24] Vapnik VN (1999). An Overview of Statistical Learning Theory. IEEE Transactions on Neural Networks.

[25] Schölkopf, B. & Smola, A. J. *Learning with Kernels* (MIT Press, 2002).

[26] Chang, C.-C. & Lin, C.-J. LibSVM: A library for support vector machines. *ACM Transactions on Intelligent Systems and Technology* **2**, 27:1–27:27 (2011). Software available at: http://www.csie.ntu.edu.tw/cjlin/libsvm.

[27] James, K. & Russell, E. Particle swarm optimization. In *Proceedings of 1995 IEEE International Conference on Neural Networks*, 1942–1948 (1995).

[28] Eberhart, Shi Y (2001). Particle swarm optimization: developments, applications and resources. Proceedings of the 2001 Congress on Evolutionary Computation.

[29] Moreira GJ, Paquete L, Duczmal LH, Menotti D, Takahashi RH (2015). Multi-objective dynamic programming for spatial cluster detection. Environmental and Ecological Statistics.

[30] Kennedy, J. & Eberhart, R. C. A discrete binary version of the particle swarm algorithm. In *Systems, Man, and Cybernetics, 1997. Computational Cybernetics and Simulation., 1997 IEEE International Conference on*, vol. 5, 4104–4108 (IEEE, 1997).

[31] Cervante, L., Xue, B., Zhang, M. & Shang, L. Binary particle swarm optimisation for feature selection: A filter based approach. In *2012 IEEE Congress on Evolutionary Computation*, 1–8 (IEEE, 2012).

[32] Chuang, L.-Y., Li, J.-C. & Yang, C.-H. Chaotic binary particle swarm optimization for feature selection using logistic map. In *Proceedings of the International MultiConference of Engineers and Computer Scientists*, vol. 1 (2008).

[33] Christov I, Bortolan G (2004). Ranking of pattern recognition parameters for premature ventricular contractions classification by neural networks. Phisyological Measurement.

[34] Özbay Y, Ceylan R, Karlik B (2006). A fuzzy clustering neural network architecture for classification of ECG arrhythmias. Computers in Biology and Medicine.

[35] Bortolan, G., Christov, I. I. & Pedrycz, W. Hyperbox classifiers for ECG beat analysis. In *Computers in Cardiology*, 145–148 (2007).

[36] Übeyli ED (2007). ECG beats classification using multiclass support vector machines with error correcting output codes. Digital Signal Processing.

[37] Minhas FA, Arif M (2008). Robust electrocardiogram (ECG) beat classification using discrete wavelet transform. Physiological Measurement.

[38] Asl BM, Setarehdan SK, Mohebbi M (2008). Support vector machine-based arrhythmia classification using reduced features of heart rate variability signal. Artificial Intelligence in Medicine.

[39] Mert A, Klç N, Akan A (2014). Evaluation of bagging ensemble method with time-domain feature extraction for diagnosing of arrhythmia beats. Neural Computing and Applications.

[40] Alickovic E, Subasi A (2015). Effect of multiscale pca de-noising in ECG beat classification for diagnosis of cardiovascular diseases. Circuits, Systems, and Signal Processing.

[41] Li, H., Yuan, D., Ma, X., Cui, D. & Cao, L. Genetic algorithm for the optimization of features and neural networks in ecg signals classification. *Scientific Reports* **7** (2017). 10.1038/srep41011 PMC5282533 28139677

[42] Soria, M. L. & Martinez, J. P. Analysis of multidomain features for ECG classification. In *Computers in Cardiology*, 561–564 (2009).

[43] Mar T, Zaunseder S, Martínez JP, Llamedo M, Poll R (2011). Optimization of ECG classification by means of feature selection. IEEE Transactions on Biomedical Engineering.

[44] Ye, C., Kumar, B. V. K. & Coimbra, M. T. Combining general multi-class and specific two-class classifiers for improved customized ECG heartbeat classification. In *International Conference on Pattern Recognition (ICPR)*, 2428–2431 (2012).

[45] Lin C-C, Yang C-M (2014). Heartbeat classification using normalized RR intervals and morphological features. Mathematical Problems in Engineering.

[46] Huang H, Liu J, Zhu Q, Wang R, Hu G (2014). A new hierarchical method for inter-patient heartbeat classification using random projections and RR intervals. Biomedical Engineering Online.

